# Teacher enthusiasm: a potential cure of academic cheating

**DOI:** 10.3389/fpsyg.2015.00318

**Published:** 2015-03-31

**Authors:** Gábor Orosz, István Tóth-Király, Beáta Bőthe, Anikó Kusztor, Zsuzsanna Üllei Kovács, Miriam Jánvári

**Affiliations:** ^1^Institute of Psychology, Faculty of Education and Psychology, Eötvös Loránd UniversityBudapest, Hungary; ^2^Institute of Cognitive Neuroscience and Psychology, MTA Research Centre for Natural SciencesBudapest, Hungary; ^3^Institute of Applied Education and Psychology, College of NyíregyházaNyíregyháza, Hungary

**Keywords:** teacher enthusiasm, academic motivations, academic cheating

## Abstract

In this research we claim that teachers’ enthusiasm matters regarding student engagement in terms of academic cheating. Previous studies found that perceived enthusiasm of teachers is positively related to the intrinsic motivation of the students. However, it was less investigated how perceived enthusiasm is related to cheating. In the first exploratory questionnaire study (*N* = 244) we found that during the exams of those teachers who are perceived to be enthusiastic students tend to cheat less. In the second questionnaire study (*N* = 266) we took academic motivations into consideration and we found that the more teachers seem enthusiastic the cheating rate will be lower among university students. Aggregated teacher enthusiasm was positively related to intrinsic motivation, negatively related to amotivation, and not related to extrinsic motivation. Aggregated teacher enthusiasm was directly and negatively linked to cheating and it explained more variance in cheating than academic motivations together. These results suggest that teachers’ perceived enthusiasm can be a yet unexplored interpersonal factor which could effectively prevent academic cheating.

The secret of genius is to carry the spirit of the child into old age, which meant never losing your enthusiasm.

Aldous Huxley

## Introduction

The word enthusiasm derives from the Greek expression *enthousiasmos* which means a divine inspiration. According to its interpretation it refers to the phenomena when a god invades someone and it fills this person’s soul with energy who becomes inspired, and who is in rapt or in ecstasy (“en” means in or into, “theos” means god). Nowadays, effective teachers are described with this characteristic. In the field of educational psychology, teachers’ enthusiasm can be approached at least in two different ways (Kunter et al., 2011): first, the behavioral approach refers to stimulating and energetic instruction practices from an external observer’s point of view as gestures, vocal delivery, or facial expressions (e.g., Collins, 1978; Sanders and Gosenpud, 1986); the second emphasizes the internal, subjective experiences (as a personal characteristic) of teachers who are enthusiastic for teaching and which deals with the teacher’s behavior as a consequence of this internal state (Kunter et al., 2011).

The diversity of the enthusiasm definition is salient. Considering ten definitions between 1970 and 2013, early authors grasp its behavioral manifestation in terms of demonstrative gestures, varied intonations, facial expressions, energetic instructions (Rosenshine, 1970; Collins, 1978; Bettencourt et al., 1983), later its positive effect on intrinsic motivation (it appears when a student engages in learning for the pleasure and satisfaction which is derived from the learning activity itself, see details below) is emphasized (Patrick et al., 2000), others describe it as an internal stable affective disposition which is linked to the motivation of the teacher. More recently, Kunter et al. (2011) focus on the internal, affective state of the teacher in terms of intrinsic motivation which promotes active involvement in teaching and leads to high quality instructional behavior. Keller (2011) similarly to early studies focuses on the behavior of teacher in terms of lively engaging presentation of class content and it appears as a personality-like characteristic which is linked to the competence and emotions of teachers. Finally, Haerens et al. (2013) conceptualize it as a positive form of involvement which goes hand in hand with the teacher engagement in warm interactions. In the present study, we intend to focus on the externally visible forms of enthusiasm instead of the subjective experience of teachers because we are afraid of social desirability biases and ceiling effect regarding self-reports of teachers concerning their enthusiasm.^1^ Despite several educational textbooks claim that one of the keys of effective teaching is enthusiasm (Wong and Wong, 2001; Stronge et al., 2004; Brophy, 2006), only a few empirical studies were carried out to measure the effect of teacher enthusiasm on students’ motivations, goals, and classroom behavior. However, all interpretation of teacher enthusiasm can have beneficial consequences concerning students’ and pupils’ learning-related emotions which are less examined in the literature of educational psychology than negative forms of learning-related emotions (Paoloni, 2014). These positive emotions can contribute both to the commitment to the task and to their stronger intrinsic motivations.

### The Effect of Teacher Enthusiasm

Rosenshine (1970) summarized the most important teachers’ enthusiasm-related studies prior to the 1970s. According to these early results, the students of those teachers who scored high on such behaviors as “stimulating”, “energetic”, “mobile”, “enthusiastic”, and “animated” have high achievements. Furthermore, the frequency of teachers’ movement, gestures, variation in voice, and eye contact were also positively related to the achievement of pupils. Following this review in the seventies, several experimental studies found that teacher enthusiasm leads to high achievements of students (Wyckoff, 1973; Williams and Ware, 1976, 1977; Land, 1980). In the following years, several studies supported these results. Teacher enthusiasm has positive effect on such outcomes as on-task behavior (Bettencourt et al., 1983), recall (Stewart, 1989), and test performance (Marlin, 1991). Not only outcomes, but motivations are also affected by teacher enthusiasm. Patrick et al. (2000) found that teacher enthusiasm is among the most important variables which are related to students’ intrinsic motivation. These results suggest not only the link between teacher enthusiasm and student outcomes, but also the causal effect of enthusiasm on achievements and motivations.

The question arises: how can teacher enthusiasm have positive effects on students’ outcomes and motivations? Keller et al. (2013) summarize three main potential mechanisms behind the positive effects of teacher enthusiasm. The first explanation reflects on the attention-commanding aspects of enthusiastic teacher behavior. According to Bettencourt et al. (1983) demonstrative gestures, varied, dramatic body movements, or uplifting vocal delivery can hold the attention of students more effectively than less enthusiastic behaviors. According to the second explanation (Frenzel et al., 2009), enthusiastic teachers become role models for their students. In this way teacher enthusiasm helps the students to adopt the teachers’ attitudes in terms of enjoyment and enthusiasm which lead to higher level of learning activity and more positive feelings toward learning (Brigham et al., 1992). The third explanation refers to the phenomena of emotional contagion (Hatfield et al., 1994). Teacher enthusiasm can be transmitted to students and it has positive effect on students’ achievement and motivation in which first, the teacher’s non-verbal communication draws the attention of students and second, she/he as a role model induces enjoyment and excitement regarding the exercises which lead to positive emotion regarding academic activities.

On the basis of the previous results enthusiasm has a positive effect on students’ achievement and their motivation which can be explained by attention drawing characteristics of enthusiastic communication, by the role-model or emotional contagion theories. However, to our best knowledge previously neither the link between teacher enthusiasm and student academic cheating, nor the relationship pattern of teacher enthusiasm and school-related general motivations were examined. The goal of this research is declaring such relationships with questionnaire methods. The goal of the first study is exploring whether teacher enthusiasm can be related to academic dishonesty of students. The second, questionnaire study aims to explore the relationship pattern between academic motivations, enthusiasm, and cheating.

### Academic Cheating

According to Brickman (1961) academic cheating reaches back to ancient times. The first recorded attempts happened in the ancient China when candidates for civil servants tried to cheat despite expected punishments for being caught were as severe as death penalty. Ehrlich et al. (1980, p. 141) defines cheating in general as behaving “dishonestly or unfairly in order to win some profit or advantage.” Garavalia et al. (2007) complement this definition with the intentionality of such behavior. Hetherington and Feldman (1964) considering the intentionality (unplanned vs. planned) of cheating made distinction between individual (i.e., using crib notes) and collaborative (i.e., whispering) forms of cheating. Furthermore, according to a further classification it is possible to distinguish plagiarism from exam cheating. While using cheating sheets or whispering are related to exam cheating, plagiarism refers to “the theft of words or ideas, beyond the point that would normally be regarded as general knowledge” (Park, 2003, p. 472). Considering such classifications in the present study, we focus on both individual and collaborative forms of intentional exam cheating.

Academic cheating is a universal phenomenon. It is present in every level of education (Anderman and Murdock, 2007). We can see a high prevalence rate among college and university students: according to the USA results (McCabe, 2005), 60% of university students cheated at least once during their academic career. Similar prevalence can be observed among South Korean (Park et al., 2013), Chinese (Ma et al., 2013), Hungarian (Orosz et al., 2013), and Western European (Teixeira and Rocha, 2010) students.

According to McCabe and Trevino (1997), two main groups of variables have effect on students’ cheating behavior: individual (e.g., achievement goals or motivations) and contextual (e.g., classroom climate or personality of teachers) factors. Previous reviews and meta-analyses (McCabe and Trevino, 1997; Whitley, 1998) suggest that contextual factors have larger impact on students’ cheating than individual factors. Therefore, we suppose that teacher enthusiasm as a contextual variable has larger impact on the cheating behavior than individual differences such as academic motivations. In the following, firstly, the effect of relevant cheating-related individual differences (academic motivations); later the effect of relevant cheating-related contextual (perceived enthusiasm) variables will be introduced.

### Academic Motivations and Cheating

On the basis of the self-determination theory (SDT) of Deci and Ryan (1985), an *intrinsically motivated* student engages in learning for the pleasure and satisfaction which is derived from the learning activity itself (Deci et al., 1991). From the side of cheating, Anderman et al. (1998), Jordan (2001), and Orosz et al. (2013) found that those students who behaved honestly in exam situations have higher intrinsic motivation than those who cheated. Furthermore, Orosz et al. (2013) results show that besides the negative link between intrinsic motivation and self-reported cheating, intrinsic motivation of high school students negatively correlated with acceptance of cheating, positively related to guilt of cheating, and with the risk of detection of cheating.

According to Patrick et al. (2000) teacher enthusiasm is among the most important interpersonal variables which can have impact on students’ intrinsic motivation: “…when a teacher exhibits greater evidence of enthusiasm students are more likely to be interested, energetic, curious, and excited about learning.” (Patrick et al., 2000, p. 233). Therefore, we may suppose that enthusiastic teaching leads to higher levels of intrinsic motivation which besides its positive effects on achievement can reduce the level of cheating.

*Extrinsic motivation* is related to those goals, which are not favorable for their pure pleasure, but for the reward and punishments coming along with the purpose (Deci and Ryan, 1985; Deci et al., 1991). Students who were intrinsically motivated to learn cheated less, while the extrinsically motivated (e.g., getting a better grade to earn a scholarship) cheated more during academic assignments (Weiss et al., 1993; Anderman et al., 1998; Murdock et al., 2001). Jordan (2001) found that higher levels of intrinsic and lower levels of extrinsic motivation resulted in high level of honesty of students during exams and assignments.

*Amotivation* occurs when one does not find relationship between his/her behavior and the experienced consequences. Therefore, the state of amotivation lacks the intention of any kind of action related to a certain area. According to some studies (Harding et al., 2004; Angell, 2006; Orosz et al., 2013; Park et al., 2013) instead of extrinsic motivation, a general lack of academic motivation, thus amotivation plays an important role in determining whether a student would behave dishonestly or not.

On the basis of these studies, we suppose that intrinsic motivation will be negatively linked to cheating and amotivation will be positively related to students’ cheating behavior. However, based on more recent studies (Orosz et al., 2013) with similar samples regarding extrinsic motivation, we expect no relationship with academic cheating.

### The Effect of Teacher Enthusiasm on Academic Cheating

According to Genereux and McLeod (1995), the personality of the teacher influences the frequency of the students’ cheating. Cheating rate is lower in the case of those teachers who are perceived as fair and friendly, who are respected by the students, and those who provide knowledge in an interesting way. Furthermore, on the basis of McCabe’s (1999) qualitative study, secondary school students are more honest during exam if they perceive their teacher is motivated, friendly, and cares about their students’ future. The opposite is true if students perceive their teacher does not care about them and their work. One of them expressed himself the following way: “A lot of the teachers that I’ve dealt with are always talking about how they can’t wait to go home… acting like they don’t want to be there. Their job is to teach me, and if they can’t do that for me, then I’m going to do what I can to move up in the world. If cheating is what I have to do, then that’s what I’m going to do.” (McCabe, 1999, p. 685). According to the results of Murdock et al. (2001) if students evaluated their teachers’ teaching competencies highly, their engagement and respect were negatively related to cheating. Cochran et al. (1999) found that most of the cheaters do not perceive the teacher competent, engaged, and as a good teacher, and they do not respect her/him. In line with these results, Genereux and McLeod (1995) also found that the interesting nature of the class, the moral engagement and the control of teacher influence students’ attitudes toward cheating.

In sum, perceived characteristics and behavior of teachers influence students’ inclination in dishonesties. If students see the teacher competent, motivated, friendly, fair, engaged, and caring, who gives interesting classes, they tend to cheat less. Furthermore, this is also true if they respect their teacher. If enthusiasm is conceptualized as stimulating and energetic instruction practices with varied vocal delivery, demonstrative gestures, large body movements, vibrant facial expression, highly descriptive word selection, and acceptance of ideas and feelings (Collins, 1978) we expect that teacher enthusiasm can similarly reduce the inclination in cheating as the above-mentioned characteristics and behaviors.

Three explanations can be taken into account concerning the reasons why teacher enthusiasm can reduce cheating behavior of students. According to the first possible explanation, the verbal and non-verbal cues of enthusiastic teaching can direct effectively the attention of students to the topics they learn (Bettencourt et al., 1983). In this way the encoding of the material requires relatively low effort which leads to better performance with reduced probability of cheating. According to the second explanation, enthusiastic teachers can become easily role models of students (Frenzel et al., 2009). If a student perceives a teacher as a role-model we can expect that the student does not want to be unfair with the appreciated teacher by cheating during an assignment. The third explanation is related to the emotion contagion theories (Hatfield et al., 1994). On the basis of this theory the intrinsic motivation of the teacher can have positive effect on the student’s interest and the heightened intrinsic motivation of the student will finally lead to decreased cheating occurrence. The present research intends to explore whether teacher’s enthusiasm can be negatively related to student cheating.

## Study 1: Teacher Enthusiasm and Academic Cheating of Students: An Exploratory Study

### Introduction

The first goal of this study was to explore whether university students can categorize most of their teachers on the basis of their enthusiastic teaching practices. Furthermore, we intended to use self-reports in order to retrospectively quantify the occurrence of cheating among the students of those teachers whose teaching practices are more or less enthusiastic. We expected that students self-report less cheating on the exams of teachers who are perceived to be more enthusiastic compared to those teachers who are perceived less enthusiastic. According to previous studies, we expect higher self-reported cheating in the case of those teachers who are characterized by less enthusiastic teaching behavior compared to those whose teaching can be characterized by more enthusiasm.

### Participants

The questionnaire was filled in by 266 Hungarian, full-time university students (152 women). The average age of the subjects was 21.48 (SD = 2.37). All of the participants were enrolled, full-time students of the University of Szeged. The study was conducted in accordance with the Declaration of Helsinki. All procedures were carried out with the adequate understanding and consent of the participants and with the approval of University of Szeged.

### Measures and Data Analysis

On the first page of the questionnaire, demographic data, such as gender and age was asked from students. Besides the two demographical questions, a 13 item scale with closed items were used which is based on the Sanders and Gosenpud (1986) questionnaire measuring enthusiasm in connection with work. The items of the survey refer to eye contact, facial expressions, gestures, body movements, word selection, vocal delivery (pitch, speech rate etc.) and general energy level. In order to adjust the questionnaire to the Hungarian higher educational context, the original items were altered and completed. In the final version, the 13 items (see **Table 1**.) represented typical teaching behaviors. Items 1–6 represented the not enthusiastic behavior of teaching, while items 7–13 represented the enthusiastic teaching behavior. Each item could grasp only one aspect of the enthusiastic teaching and in this way we cannot claim that single items can be reliable and valid indicator of teacher enthusiasm as a whole. The respondents first decided whether they had a university lecturer from last year who can be characterized by these given behaviors (they were instructed to refer to the most typical teacher in case they had more than one) and then they were instructed to answer whether they cheated during the given teacher’s exam. Therefore, students could think of maximum 13 different teachers. We predefined cheating to the students as a behavior which includes using cheat-sheets, copying, whispering, plagiarism, submitting the same script in different courses, using unauthorized electronic equipment, assuming another individual’s identity during an exam or handing in an essay created by another person. With this data gathering method we intended to create a questionnaire which can be filled in quickly (less than 5 min) and without a lot of effort from the part of the students. Furthermore, we intended to explore whether students report less cheating concerning the exams of those teachers whose teaching behavior can be characterized by a special aspect of enthusiasm (items 7–13) compared to those teachers whose teaching activity can be described by a special aspect of non-enthusiasm (items 1–6).

**Table 1:** Descriptive data concerning the teacher enthusiasm and self-reported cheating of students.

Items	Percentage of students who had a teacher who can be characterized with the description (%)	Percentage of students who cheated during the exam of this teacher (%)
(1) Standing or sitting in one place during all the course	86.1	66.9
(2) Reading booklets or slides	83.5	56.8
(3) Speaking in simple dialogs, rarely using similes and metaphors, explaining dimly	73.7	55.3
(4) Lethargic, inert, depressed, seems sleepy, and tired	74.4	48.1
(5) Not gesticulate or cramped, making clumsy movements at courses	71.8	28.2
(6) Expressionless face, a little musty or gloomy	78.2	18.8
(7) Dynamic and usually speaks by heart	94.4	15
(8) Maintains eye-contact while avoiding staring, pays attention to student’s reactions	96.6	13.5
(9) Large demonstrative movements, rapid, energetic and natural movements and raises volume to emphasize	89.1	13.5
(10) Highly varied tone, pitch, volume and cadence, excellence articulation, variations from rapid excited speech to whisper	70.3	13.5
(11) Highly descriptive, excellent and frequently uses similes and metaphors	84.6	13.2
(12) Energetic, drive and spirit throughout sessions, inspiring	87.6	13.2
(13) Shining face, plays with mimicry and gestures, smiles a lot	82.7	9.4

### The Process of Data Gathering

The participants were informed about the purpose of the research personally by Psychology BA students. The paper-and-pencil anonymous measurement was voluntarily filled out at Klebelsberg Library of the University of Szeged, the respondents did not get any compensation for the participation. They were ensured that their responses will be kept confidential. Besides the verbal information, the questionnaire indicated that it is fully voluntary and anonym. The subjects were asked to be as honest as possible to make sure we get authentic results. The average time to fill out a questionnaire was 5 min.

### Results

The 266 participants could use most of our descriptions for one of their former teachers. For details see **Table 1**. The lowest proportion of students (70.3%) could remember a teacher who highly varied tone, volume, and excellence articulation, variation from rapid excited speech to whisper. Moreover, the largest proportion of students (96.6%) could remember a teacher who maintained eye contact, paid attention to the students’ reactions, and did not stare. Overall, in every case more than 70% of the students could characterize at least one teacher on the basis of our 13 descriptions which are related to enthusiastic teaching practices. Furthermore, **Table 1** provides information concerning whether students cheated or not during the teacher’s exams who is characterized by our descriptions. The fewest number of students (9.4%) reported cheating if the teacher’s teaching practice could be characterized by shining face, playful mimicry and gestures, and a lot of smiles. However, when the teacher’s instructional practices were described as passive in terms of standing or sitting in one place during the whole course, almost 70% of the students reported exam cheating. Similarly high (56.8%) proportion of respondents reported cheating in cases of those teachers who read booklets or slides during the lecture. Participants reported relatively high cheating rate in case of those teachers who spoke in simple dialogs, rarely used similes and metaphors and explained dimly (55.3%); and in the case of those who were lethargic, inert, depressed, sleepy, and tired (48.1%). Concerning self-reported cheating rate, there is a larger gap between the above mentioned items and the fifth and sixth items (Not gesticulate or cramped, making clumsy movements at courses, 28.2%; Expressionless face, a little musty or gloomy, 18.8%). Contrasting to the first six items, cheating rate was between 9 and 15% in the case of the all of five enthusiastic behavior items. In sum, most participants could find a teacher who can be characterized by these enthusiasm dimensions, and visible differences appeared in terms of cheating between students of those teachers who are characterized by enthusiastic traits compared to those who are described as rather lethargic.

### Discussion

The first descriptive study had two main results. First, students can retrospectively categorize teachers on the basis of the enthusiasm dimension of teaching practices. Second, the results suggest that teacher enthusiasm matters in terms of academic cheating. Seven times more students reported exam cheating in the case of those teachers who are standing or sitting during the whole course compared to those teachers who play with mimicry and gestures and who smile a lot with a shining face. But four times more cheating was reported during the exams of those teachers who read books and slides compared to those who are highly descriptive, excellent, and use metaphors.

Although the altered version of Sanders and Gosenpud (1986) measurement appears to be adequate for describing teachers’ enthusiastic behavior, this study has several limitations. First of all, it is a self-report study which is based on memory recollection. These memories can be distorted over time. Second, students were asked to respond in a dichotomous scale while describing the teachers and regarding cheating, as well. A more refined continuous measurement would allow refined statistical analyses and it would allow the examination of the factor structure of the used enthusiasm scale. Third, in this case they could report cheating behavior in a generalized way including plagiarism, individual, and collaborative exam cheating, etc. A more detailed measurement could be useful for further analyses. Fourth, the used sample was not representative in any respect. Fifth, we did not measure the mediating effect of any potential variables.

The following study will challenge some of these limitations. Namely, Study 2 will provide the possibility to measure different forms of self-reported cheating behavior in a continuous and not in a dichotomous way. It allows in-depth statistical analyses regarding both internal structure of the used scales and their relationship patterns. Furthermore, in Study 2, students are allowed to estimate the proportion of their teachers who can be described with the enthusiastic behaviors above mentioned. Finally, in Study 2, we can explore the mediating effect of academic motivations between perceived aggregated teacher enthusiasm and self-reported cheating.

### Conclusion

This study showed that teacher enthusiasm appears to be a relevant instructional behavior in reducing cheating behavior of students which requires further, in-depth correlational and experimental examination.

## Study 2: The Link between Teacher Enthusiasm, Academic Motivations and Academic Cheating

### Introduction

Several studies investigated the relationship between cheating and academic motivation. Regarding teacher enthusiasm, it is considered to be one of the most important aspects of teaching (Brophy and Good, 1986). Patrick et al. (2000) found that by being enthusiastic, teachers can enhance students’ intrinsic motivation. Moreover, if students considered their classes interesting (which is one of the cornerstones of intrinsic motivation), they cheated less (Pulvers and Diekhoff, 1999). Orosz et al. (2013) also concluded that intrinsic motivation can greatly reduce the possibility of cheating. Therefore, it would be possible to reduce the amount of academic dishonesty by putting emphasis on the intrinsic value of learning instead of emphasizing the importance of good grades (Tibbetts, 1999; Meece et al., 2006).

The main goal of the present study is to investigate whether teachers’ perceived enthusiasm or academic motivation directly or indirectly—through academic motivations—influences students’ self-reported academic cheating. On the basis of the studies mentioned above, we assume that aggregated teacher enthusiasm can reduce cheating through enhancement of the interest and intrinsic motivation of students or it might be possible that it has a motivation independent effect also on students’ cheating behavior. If a student has a lot of enthusiastic teacher the frequency of cheating is lower considering all exams at the end of the semester. Enthusiastic teachers can create a more stimulating teaching environment (Kunter et al., 2008) and the verbal and non-verbal cues of the teaching behavior can direct the attention of the students to the given subject (Bettencourt et al., 1983) and it might be possible that students can more easily encode the material during the class, thus they will need less efforts and learning in order to resolve exam exercises which can finally lead to lower cheating rates. Furthermore, it is also possible that enthusiastic teachers are perceived as role models by the students (Frenzel et al., 2009) and students do not want to be unfair with their role models by cheating during the assignment of someone they highly appreciate. If a student has several enthusiastic teachers (s)he does not intend to be unfair with these teachers in terms of cheating. However, if we consider the emotional contagion theories (Hatfield et al., 1994), it might be possible that the intrinsic motivation of teachers raises the interest of students in a given material and this way the intrinsic motivation will mediate between teacher enthusiasm and lower cheating rates. Consequently, students will make more efforts and spend more time with learning if they have a lot of enthusiastic teachers and as a consequence of this behavior, students will cheat less as a whole at the end of the semester.

First, (H1) we assume that if a student has a lot of teachers who are perceived to teach enthusiastically this higher proportion has a direct negative effect on academic dishonesty. Such teachers orient the attention of students during class activities (Bettencourt et al., 1983), hence students can more deeply encode the materials during class and they have to make less effort after school in order to learn the material. Another explanation for the direct negative link between aggregated teacher enthusiasm and student cheating is related to the more salient unfairness of cheating at a class with a teacher who is a role model due to her/his enthusiastic teaching behavior compared to others who are not perceived as role models (Frenzel et al., 2009).

Besides the direct relationships, (H2) we suppose that aggregated teacher enthusiasm (high proportion of enthusiastic teachers) can influence students’ cheating behavior indirectly through their academic motivations. Previous studies found that higher intrinsic motivation reduces cheating, while amotivation increases it. In the present study we expect this relationship pattern (H2a). Regarding the link between students’ cheating and extrinsic motivation, previous results are more ambiguous. In line with previous Hungarian results (Orosz et al., 2013), we expect no relationship between extrinsic motivation and cheating (H2b).

Considering the emotional contagion explanations, if the teacher is enthusiastic, the students’ intrinsic motivation will increase (Hatfield et al., 1994) which will lead to lower cheating rates (Weiss et al., 1993; Anderman et al., 1998; Murdock et al., 2001; Orosz et al., 2013). We expect this mediated relationship pattern (H2c). However, to our best knowledge no prior results were found regarding the link between amotivation of students and teacher enthusiasm. However, we expect a negative link between amotivation and perceived and we expect that amotivation is positively related to students cheating based on the results of previous studies (Harding et al., 2004; Angell, 2006; Orosz et al., 2013; Park et al., 2013). Therefore, we expect this mediated relationship pattern (H2d).

### Materials and Methods

#### Participants

Three hundred and forty two university students (*M* = 224, *F* = 116) participated in the study. The respondents’ age was between 18 and 41 years; the average age was 22.11 (SD = 2.53). The students’ GPA was 4.03 (SD = 0.70) in the previous semester. Regarding the education level of parents, 6.2% of mothers have a primary level of education, 48.2% the secondary-level, 42.9% have a college or university degree, while 2.6% of the mothers have other qualifications. Regarding the fathers, 5.6% have a primary level of education, 57.6% a secondary-level, 34.1% have a higher-education degree, while 2.6% of the fathers have other qualifications. Participants were informed about the content of the questionnaire, e.g., academic motivation, teachers’ enthusiasm, and academic dishonesty. Respondents volunteered for the study and did not receive compensation for participation; moreover, students were assured of their anonymity. The research was conducted with an online questionnaire, filling this out lasted approximately 10 min, students were asked to respond as honestly as possible.

#### Variables and Measures

The first page of the questionnaire included demographic data regarding age, gender, qualifications of parents, and GPA from their last semester. The next section was intended to measure the academic motivation of the students. We used the Hungarian version (Orosz et al., 2013) of Vallerand et al.’s (1992) Academic Motivation Scale (AMS) for university samples. This scale was created to measure academic motivation at contextual level. The items appear as answers to the following question: Why do you go to college? This shorter version of AMS contained three factors: amotivation, extrinsic, and intrinsic motivation. The response choices for these items were rated on a 7-point Likert scale (1 = does not correspond at all; 2–3 = corresponds a little; 4 = corresponds moderately; 5–6 = corresponds a lot; and 7 = corresponds exactly). The reliability in terms of internal consistency was acceptable (α_intrinsicmotivation_ _to_ _know_ = 0.86; α_extrinsicmotivationexternalregulation_ = 0.80; α_amotivation_ = 0.87).

The second questionnaire measured academic cheating. We used the slightly modified Hungarian version of McCabe and Trevino’s (1993) Academic Dishonesty Scale, which contained 10 items. The respondents had to rate each item using a 5-point Likert scale (1 = ”never”; 2 = ”one or two times”; 3 = ”three–five times”; 4 = ”six–ten times”; 5 = “more than 10 times”) and had to indicate how many times they used different forms of cheating in the previous semester. The reliability in terms of internal consistency was acceptable (α_cheating_ = 0.84).

In the next section, we aimed to measure perceived aggregated enthusiasm of teachers using Sanders and Gosenpud’s (1986) work-enthusiasm questionnaire, which contains 13 items. We used the same items as in the Study 1 (see **Table 1**.). However, students rated differently these items: they were instructed to indicate on an 11-point scale that out of 10 teachers how many of their teachers can be characterized on the basis of each item concerning their enthusiastic teaching behavior during class (0 – none of them, 1 – 1 out of 10, etc.). Therefore, in this case we measured the proportion of teachers concerning each item they have by using the modified version of Sanders and Gosenpud’s (1986) enthusiasm scale—interpreting it as an aggregated perceived enthusiasm. This sort of measurement can allow the investigation of the relationship pattern of the enthusiasm items (i.e., if a student has a lot of enthusiastic teachers she/he will give consequently higher scores in the case of items 7–13 and lower scores in the case of items 1–6). We chose this unusual form of measurement for two reasons. First, the AMS grasps academic motivations (Vallerand et al., 1992) and the McCabe and Trevino Academic Dishonesty Scale (McCabe and Trevino, 1993) grasps cheating in a contextual, school level (see Vallerand and Ratelle, 2002). This contextual level means that students fill out the questionnaire concerning not specific classes but at the level of school. Concerning AMS students reply to the question of AMS “Why do you go to school?”; concerning the used cheating scale they report overall cheating concerning last semester. Dissimilarly to previous studies (Kunter et al., 2008, 2013) we did not intend to ask teachers’ self-reports concerning their own enthusiasm (see Footnote 1). Furthermore, dissimilarly to other research (Patrick et al., 2000) we did not intend to ask students perceived enthusiasm at a lower, more situational level by asking the perceived enthusiasm of specific teachers during specific courses because it would have been incompatible with the measurement level of both the AMS and the McCabe and Trevino’s cheating measure. The second reason is the following. If we measure teacher enthusiasm at a course-specific (or teacher-specific) level we should also do the same with the motivations and the cheating. We were afraid of the lack of reliability of the student responses if we ask them about their course specific cheating. Very probably, students would be very suspicious if they are asked concerning whether they turned in work done by someone else concerning their Introduction to psychology course. Despite cheating occurrence per semester is high in Hungary (Orosz et al., 2013), very probably if cheating is asked concerning specific courses, problems of social desirable responding would appear in a much larger extent compared to the case if students are asked about their aggregated frequency of cheating concerning their last semester. In the case of this sort of measurement the reliability in terms of internal consistency was high regarding both enthusiastic items (α_items7-13_ = 0.92) and non-enthusiastic items (α_items1-6_ = 0.86).

The study was conducted in accordance with the Declaration of Helsinki. All procedures were carried out with the adequate understanding and consent of the participants and with the approval of Eötvös Loránd University.

#### Data Analysis

The statistical analyses were performed by the SPSS version 15 and Amos version 17. Path analyses were conducted on covariance matrices, and the solutions were generated by ML estimation. Based on Brown (2012), several goodness of fit indices were included: chi-square degree of freedom ratio (chi-square/df), comparative fit index (CFI), the Tucker-Lewis index (TLI), and the root mean square error of approximation (RMSEA). Following Hu and Bentler’s (1999) suggestions, acceptable model fit was defined by the following criteria: CFI (≥0.95), TLI (≥0.95) and RMSEA (≤0.06).

### Results

To our best knowledge, no previous studies examined the joint impact of teachers’ enthusiasm and students’ academic motivation in relation to academic cheating. Therefore, we intended to explore how teachers’ enthusiasm and the different types of academic motivation are connected to students’ academic cheating. Moreover, we intended to investigate the direct and indirect influence of the observed variables. We expected, on the basis of the correlations and the theoretical background, that the exploratory path model would reveal (1) the direct effect of teachers’ enthusiasm and lack of enthusiasm on students’ academic cheating behavior, and (2) the indirect effects of aggregated teacher enthusiasm and lack of enthusiasm through students’ academic motivation to students’ academic dishonesty.

Structural equation modeling (SEM) was used to explore the relationship pattern of academic cheating, students’ academic motivation, and the perceived aggregated enthusiasm of teachers. Parcels were used as indicators of academic cheating and aggregated teacher enthusiasm, because the variables contained too many items. We found justifiable using parcels because the scales (academic cheating and enthusiasm) were theoretically unidimensional (Bandalos and Finney, 2001). Furthermore, previous studies used this method can be used in the case if there are several latent variables (i.e., Carbonneau et al., 2008).

We used factorial algorithm on the basis of Rogers and Schmitt (2004). In this algorithm we computed parcels on the basis of exploratory factor analysis which resulted in factor loadings. In the case of both the academic cheating measure and the enthusiasm measure each parcel sequentially took up the items with the highest to the lowest factor loadings by alternating the direction of item-choosing turns through the parcels. For cheating, we aggregated Items 6 and 3 into Parcel 1, Items 9 and 7 into Parcel 2, Items 10 and 2 into Parcel 3, Items 5 and 8 into Parcel 4 and Items 4 and 1 into Parcel 5. The teachers’ enthusiasm variable contains two components: one of the components refers to teachers’ enthusiastic behavior; the other refers to teachers’ non-enthusiastic behavior. For the enthusiasm component, we aggregated Items 9 and 7 into Parcel 1, Items 12 and 8 into Parcel 2 and Items 13, 10 and 11 into Parcel 3. For the non-enthusiasm component, we aggregated Items 5 and 1 into Parcel 1, Items 4 and 2 into Parcel 2 and Items 3 and 6 into Parcel 3. The non-enthusiastic component was represented more (β = 0.56, *p* < 0.001) in the total teachers’ enthusiasm factor, while the enthusiastic factor explained a smaller amount of the variance of it (β = –0.32, *p <*0.001).

**FIGURE 1:**
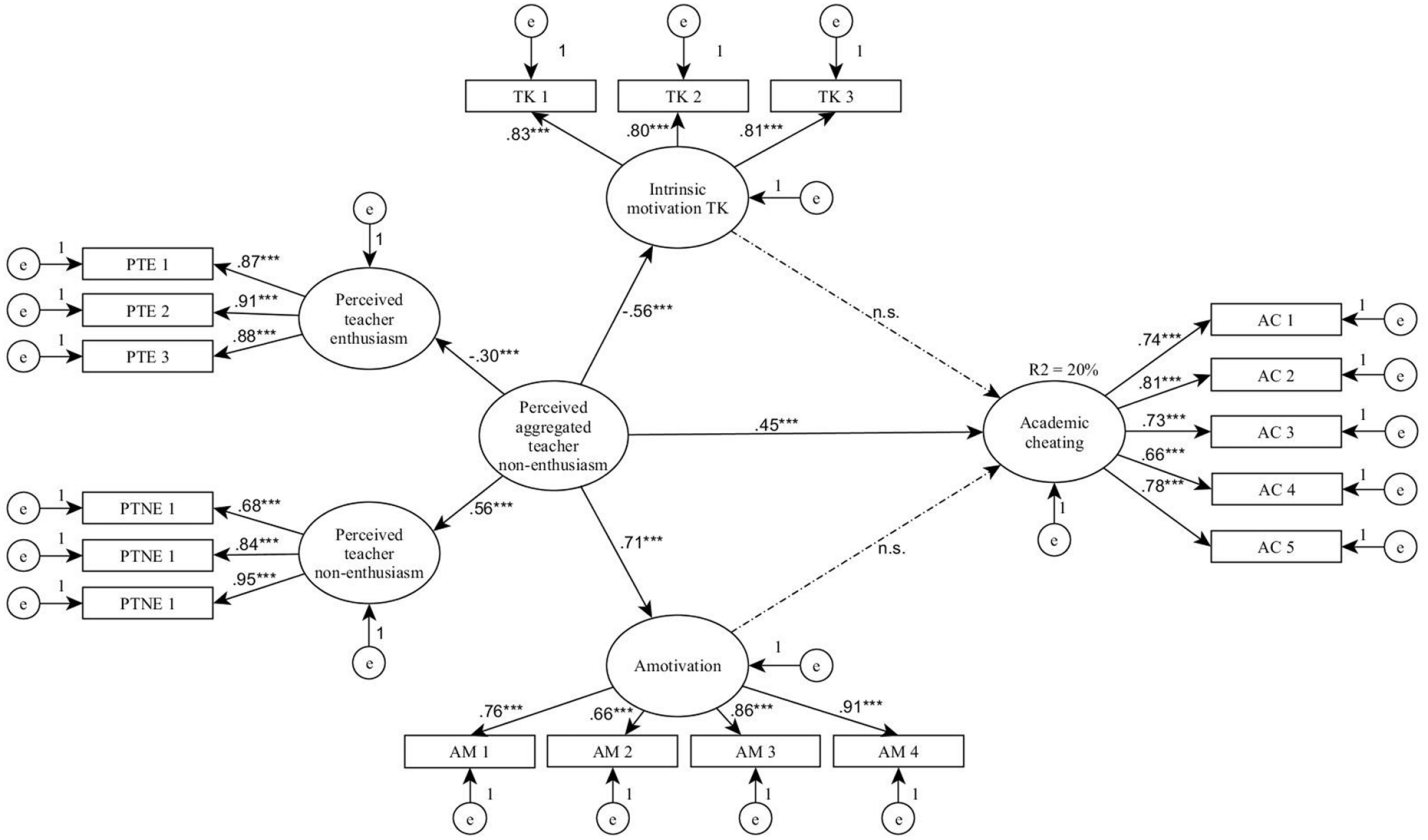
Results of the structural equation modeling. Statistical significance: ^∗∗∗^*p* < 0.001; n.s., not significant result; TK, to-know; AM, amotivation; PTE, perceived teacher enthusiasm; PTNE, perceived teacher non-enthusiasm; AC, academic cheating.

Several models were tested.^2^ Here, only the final best fitting model is presented in **Figure 1** with standardized estimates. According to the final model [χ^2^ (130, *N* = 340) = 309.635, *p* < 0.001 (χ^2^/*df*= 2.382), CFI = 0.948, TLI = 0.939, RMSEA = 0.064], only lack of teachers’ perceived enthusiasm (β = 0.45, *p*< 0.001) had direct effect on self-reported academic cheating (*R*^2^= 20.0%). Furthermore, lack of teacher enthusiasm had a direct effect on both intrinsic motivation (β = –0.51, *p*< 0.001) and amotivation (β = 0.67, *p*< 0.001). However, neither motivation mediated the effect of teacher enthusiasm concerning students’ cheating. These results are in line with our first hypothesis (H1): namely, teachers’ perceived enthusiasm has a direct effect on self-reported cheating. However, the second hypothesis (H2) was not confirmed because neither intrinsic motivation, nor amotivation had a direct effect. Furthermore, in line with previous results, extrinsic motivation was not linked to cheating and it was also unrelated to teachers’ enthusiasm. In sum, the more teachers are perceived enthusiastic the less cheating is reported among university students.

### Discussion

This exploratory study investigated whether lack of teachers’ perceived enthusiasm could have an effect on academic cheating. Our results suggest that without the mediation of academic motivations, the number of teachers who are perceived to be enthusiastic is negatively related to cheating rates. This effect could have multiple explanations. First, based on Frenzel et al.’s (2009) explanation, we can assume that enthusiastic teachers can serve as role models for students. Therefore, when students decide about cheating or being honest during an assignment, they are more likely to choose honesty, because they might consider cheating against that teacher unfair. If they have a lot of enthusiastic teachers they will cheat less during the exams. Another possible explanation is that teachers with enthusiastic behavior can orient the attention to the material more easily (Bettencourt et al., 1983), thus the students can learn it during class. If they can learn the material more easily thanks to the enthusiastic teaching behavior of the teacher, they have to study less for the exam (they have to make less effort) in order to get an appropriate result, which can finally lead to lower cheating rate.

Our second goal (H2) was to identify the possible indirect effects of the lack of teachers’ enthusiasm on students’ academic cheating behavior through the academic motivations of the students^3^. Interestingly, (H2a and H2b) no link has been found between the different types of motivation and self-reported academic cheating. Orosz et al. (2013) found that extrinsic motivation is not related to cheating, whereas intrinsic motivation and amotivation correlated with cheating. Harding et al. (2004), Angell (2006), and Park et al. (2013) also found that amotivation is an important predictor of cheating. Our analysis confirms that extrinsic motivation does not have any influence on cheating which means that the relationship between the two constructs does not appear to be as clear as previous studies (Weiss et al., 1993; Anderman et al., 1998; Murdock et al., 2001) have found. However, contrary to previous results (Orosz et al., 2013), we found that neither intrinsic motivation, nor amotivation had a significant direct effect on academic cheating if we include teacher enthusiasm in the same model. The consensus regarding links between academic motivations and academic cheating seems to be widespread (Anderman and Murdock, 2007). However, aggregated teacher enthusiasm might be one of the important background interpersonal variables behind intrapersonal motivations such as amotivation or intrinsic motivation. This result is in line with McCabe and Trevino’s (1997) claim that contextual elements are more important than individual ones in the context of academic cheating and it is also compatible with Whitley’s (1998) meta-analysis which pointed out that individual factors are less important than interpersonal, situational predictors.

On the other hand, (H2c and H2d) teachers’ lack of enthusiasm had a negative direct effect on intrinsic motivation and a positive direct effect on amotivation. These results can be explained by the emotional contagion theories (Hatfield et al., 1994). According to our results encountering a lot of enthusiastic, intrinsically motivated teachers can raise the interest of the students in a given material and this way it increases their intrinsic motivation. However, besides the motivation-related beneficial effect of teacher enthusiasm, cheating is not only decreasing because of the heightened intrinsic motivation but also other reasons which require further investigations.

It is important to note that similarly to other research, this one also has limitations. First, we have to mention that it is a cross-sectional study. Respondents had to answer through the Internet which can always raise questions about the real identity of the respondent. However, on the other side, it can reduce the social desirability bias. Second, we measured only one type of mediator variable in terms of academic motivations. However, further studies should explore other relevant teaching-related variables. Teacher enthusiasm may involve diverse teacher characteristics. In order to separate these characteristics from teacher enthusiasm it would be fruitful to discriminate enthusiasm from such behaviors. One of these characteristics might be mastery-oriented teaching. In further studies it would be important to measure separate effect of perceived mastery-oriented teaching from teacher enthusiasm. Moreover, we have no information about the incompletion rate. Students of different institutions have participated in this research but the sample is not representative to the country as it only includes university students, but not elementary and high school students. It also has to be mentioned that both the AMS and the scale measuring teachers’ enthusiasm might need further evaluation and examination.

Furthermore, different forms of cheating could be separated in future studies, for instance copying from other students, plagiarism or usage of cheat sheets. It would be useful to reveal what other teacher-related factors—both individual and contextual—could possibly influence students’ academic dishonesty and how they exert their influence on this variable. Furthermore, it is possible that not only very visible forms of enthusiasm matter. Our scale included very salient behavioral forms of teacher enthusiasm. However, more tacit cues could be as important as the visible ones.

### Conclusion

The lack of teacher enthusiasm appears to diminish the effect of academic motivations on students’ self-reported cheating behavior. Simultaneously, relatively strong negative link was found between the number of not enthusiastic teachers and the students’ intrinsic motivation and similarly strong positive link was found between amotivation and the low number of enthusiastic teachers. Consequently, this study showed that if a student has a very few enthusiastic teachers, (s)he will not only have reduced intrinsic motivation, but (s)he might more easily become amotivated. Besides all of these negative consequences, these students also report higher cheating rate. These results are in line with previous reviews and meta-analyses (McCabe and Trevino, 1997; Whitley, 1998) which claim that situational and interpersonal variables are more important regarding student cheating than individual differences such as academic motivations.

## General Discussion

Many predictors of academic cheating were explored previously (Whitley, 1998). These predictors can be categorized into three main categories: individual differences, situational and interpersonal variables, and cultural effects (Orosz, 2010). During several years among intra-individual variables academic motivations and academic goals were examined as key predictors of cheating (Anderman and Murdock, 2007). According to Whitley’s (1998) meta-analysis, situational and interpersonal variables appeared to have the largest effect on cheating. Among these variables we can emphasize the role of teacher who creates a given context in which cheating can appear. Besides individual differences and situational (interpersonal) variables, only a few empirical studies examined systematically the effect of culture on cheating behavior (Grimes, 2004; Teixeira and Rocha, 2010). These broader societal-level value-related variables can also influence the situational level, and consequently the behavior of teacher, which provides the proximal context of cheating. In the first study, on the basis of self-reports we found that teacher enthusiasm as a situational (interpersonal) variable matters in academic cheating.

Previous studies showed that the student–teacher relationship has to be taken into account in relationship with academic cheating. Students cheat less if the teacher is perceived to be friendly, motivated, engaged, and who gives an interesting lecture (Genereux and McLeod, 1995; McCabe, 1999; Murdock et al., 2001). If we take a closer look on the Enthusiasm items generated on the basis of Sanders and Gosenpud (1986) the dimension of friendliness (including reversed items) appears in items 3(R), 4(R), 6(R), 8, 11, and 13; motivation or its opposite appears in items 4(R), 6(R), 7, 9, 12, and 13; engagement appears in all items, and interesting lecture appears in items 3(R), 7, 8, and 11. Therefore, teacher enthusiasm can be a compound of these previously explored variables which has negative effect on cheating.

All of the above-mentioned teacher characteristics and behavior is linked to students’ academic motivation and their academic goals. Previous studies found that intrinsic motivation and mastery goal orientation negatively related to cheating (Weiss et al., 1993; Anderman et al., 1998; Pulvers and Diekhoff, 1999; Wryobeck and Whitley, 1999; Jordan, 2001; Anderman and Murdock, 2007). Whereas, some studies showed that extrinsic forms of motivation are positively related to cheating (Anderman and Murdock, 2007), others—similarly to the present results (H2b)—found no link between cheating and extrinsic motivation (Orosz et al., 2013). Only a few study examined the link between amotivation and cheating and they found positive link between this form of motivation and self-reported cheating (Orosz et al., 2013). As Anderman and Murdock (2007) showed that student motivations do not exist in a vacuum, but in a certain classroom climate. This climate can be affected fundamentally by the enthusiasm of the teachers. Study 2 showed that the more enthusiastic teachers students have, the less they cheat (H1). Furthermore, if students see the most of their teachers enthusiastic they are intrinsically motivated (H2a) and they are not amotivated (H2a). Therefore, in line with previous results (Patrick et al., 2000) teacher enthusiasm is among the most important variables which are linked to students’ intrinsic motivation: the number of enthusiastic teachers explains a relatively large amount of variance of both intrinsic motivation and amotivation.

However, on the basis of the results of Study 2 we could see that link between aggregated teacher enthusiasm and academic cheating is not mediated by either intrinsic motivation (H2c) or by amotivation (H2d). (Neither cheating, nor aggregated enthusiasm was related to extrinsic motivation.) In the present model, the lack of link between motivation and cheating can be attributed to a background variable (overall enthusiasm of teachers) which appears to be one of the important sources of student motivation, and which diminishes the link between motivations and cheating. The question arises: what can be the mechanism through perceived aggregated enthusiasm explains directly 20 percent of the variance of students’ cheating. Keller et al. (2013) mention three main explanations: the attention commanding one (Bettencourt et al., 1983), the role-model one (Frenzel et al., 2009), and the enthusiasm contagion one (Hatfield et al., 1994). The first and second studies can hardly define the unique role of these explanations. Taking into account the content of enthusiasm items, enthusiastic teachers can draw the attention of students, they can be role models, and the contagion can also occur. Possibly, all of these effects can simultaneously lead to less cheating. Besides these effects it is possible that enthusiastic teachers behave differently not only during courses but during exams compared to less enthusiastic teachers. It is possible that enthusiastic teachers survey more carefully exams, update the exam questions year by year, ask more questions during exams which need more thinking and less material to memorize, and they can create other ways exam situations (by providing sitting order) in which students can hardly cheat. In sum, it is possible that enthusiastic teachers do their best not only during class but during exams, as well. Besides these explanations it is possible that enthusiastic teachers are also perceived as teachers who make a lot efforts in order to do their best during classes (and exams), students might feel unfair cheating against some who does his/her job conscientiously. In sum, further studies are needed in order to separate how the above mentioned mechanisms of teacher enthusiasm influence cheating. The background variable behind motivations can be the teacher enthusiasm. However, it is possible to suppose that behind perceived enthusiasm exam-related specific behaviors can be supposed which can be explained by “classical” interpersonal variables as risk of detection, expected punishment, or sitting order, etc.

It was an Eastern-European research. According to previous results (Grimes, 2004; Teixeira and Rocha, 2010; Orosz et al., 2013) in Eastern-European countries cheating rates are higher compared to other Western European countries, US students and Asian students. Therefore, potential variables which have impact on academic cheating can be culture-specific. Maybe in other cultures perceived enthusiasm has smaller or larger effect on cheating than in the Eastern-European context. Further cross-cultural examination is needed in order to explore these effects. Maybe perceived teacher enthusiasm can be dissimilar in different cultures which is linked to the teachers’ own evaluation on their societal-level reputation or socioeconomic status. Maybe in such countries where teachers are less overloaded, they appear to be more enthusiastic than in countries in which they have more compulsory work.

Besides the culture-specificity, several limitations can be mentioned regarding the two studies. The first study was based on self-reports, it was based on perceived enthusiasm which derives from the evaluation of students. In this study one item indicated only one teacher. Furthermore, their responses relied on their memory recollection from last year. There might be distortion concerning the details. Despite the potential distortions, it seems that the magnitude of self-reported cheating occurrence differences were visible. Finally, the first study was descriptive which without in-depth statistical analysis provided only guidelines for further research. The second study was also based on self-reports. Regarding the path model, in the analysis we used parcels in order to reduce the complexity of the model. We used the three factor version of Vallerand et al.’s (1992) the AMS which showed good model fit in the case of previous Hungarian studies (Orosz et al., 2013). Similarly to the first study, we did not have data from self-perception of teachers which could allow measuring the discrepancies between how students perceive their teachers enthusiasm and how teachers perceive their own enthusiasm. We did not measure either in the first or in the second study directly the cheating behavior of the students. Finally, study two provided aggregated information concerning both several teachers and several forms of teaching. This study does not provide information concerning the effect of specific forms of cheating during the exams of specific teachers, but an overall evaluation in terms of perceived enthusiasm climate and various forms of cheating.

The present research has two main practical implications. If students have many enthusiastic teachers they cheat less. Furthermore, similarly to other studies, perceived aggregated teacher enthusiasm is positively related to intrinsic motivation and negatively related to amotivation. Consequently, it is important to keep teachers enthusiastic concerning their subject and instructional activities. It is especially true if we keep motivated the whole teaching staff. Despite its powerful positive impact, to our best knowledge no prior study examined the long term effect of enthusiasm specific interventions.

## Conclusion

The present research aimed to measure the effect of teacher enthusiasm on self-reported cheating. The results suggest that aggregated teacher enthusiasm is related to self-reported academic cheating independently from motivations (intrinsic motivation, extrinsic motivation, amotivation). The questionnaire results support the main conclusion which is enthusiasm matters in cheating. However, the underlying mechanisms through which enthusiasm reduce cheating is still unexplored. Further studies are required in order to support the relevance of the three main (and other alternative) hypothesis (attention command, role-model, contagion). Further research is needed to explore the further potential positive effects of enthusiasm interventions among teachers and other professionals.

## Footnotes

1 According to a preliminary study we found significant relationship between self-reported enthusiasm of social desirability regarding a Hungarian high-school teacher sample (*N* = 188) by using Kunter et al.’s (2008) Teacher Enthusiasm Scale and the B shortened version (Reynolds and Gerbasi, 1982) of Marlowe Crowne Social Desirability Scale (1960). This social desirability scale correlated relatively strongly with Kunter et al.’s (2008) both subject enthusiasm scale (*N*_items_ = 5, 7-point Likert scale, 1 = not at all true, 7 = completely true) referring to topic-related affected orientation which involves enthusiasm toward the material (*r*= 0.316, *p* < 0.001) and teaching enthusiasm (*N*_items_ = 5, 7-point Likert scale, 1 = not at all true, 7 = completely true) referring to the enjoyment, pleasure and enthusiasm regarding the teaching activity (*r*= 0.421, *p* < 0.001). Furthermore, in the case of enthusiasm means—contrary to Kunter et al.’s (2008) results —ceiling effect was measured (*M*_subjectenthusiasm_ = 6.5, SD_subjectenthusiasm_ = 0.54; *M*_teachingenthusiasm_ = 6.65, SD_teachingenthusiasm_ = 0.49).

2 Alternative models are available upon request.

3 As noted previously, it is possible that with their enthusiastic behavior, teachers can increase students’ intrinsic motivation (Patrick et al., 2000). Moreover, students with increased intrinsic motivation are likely to cheat less than their extrinsically motivated or amotivated classmates (Anderman et al., 1998; Murdock et al., 2001; Orosz et al., 2013).
